# Evaluating machine learning models for stroke prediction based on clinical variables

**DOI:** 10.3389/fneur.2025.1668420

**Published:** 2025-09-12

**Authors:** Patrick O. Akinwumi, Stephen Ojo, Thomas I. Nathaniel, James Wanliss, Olukayode Karunwi, Mercy Sulaiman

**Affiliations:** ^1^College of Education, Clemson University, Clemson, SC, United States; ^2^College of Engineering, Anderson University, Anderson, SC, United States; ^3^School of Medicine Greenville, University of South Carolina, Columbia, SC, United States; ^4^College of Arts and Sciences, Anderson University, Anderson, SC, United States; ^5^Department of Psychology, University of New Hampshire, Durham, NH, United States

**Keywords:** stroke risk prediction, machine learning in healthcare, clinical decision support systems, predictive modelling, feature importance analysis, imbalanced data handling

## Abstract

**Introduction:**

Stroke remains one of the leading causes of global mortality and long-term disability, driving the urgent need for accurate and early risk prediction tools. Traditional models such as the Framingham Stroke Risk Score have provided foundational insights into stroke prevention but are constrained by linear assumptions and limited adaptability to complex real-world data. In contrast, machine learning (ML) techniques offer the ability to model non-linear relationships and interactions among diverse clinical and demographic variables, supporting more personalized and flexible risk prediction.

**Methods:**

This study evaluates five supervised ML algorithms, Logistic Regression, Random Forest, Gradient Boosting, Support Vector Machine (SVM), and K-Nearest Neighbours (KNN), using a publicly available dataset from Kaggle. Following class imbalance correction, models were assessed using multiple metrics including accuracy, ROC-AUC, and confusion matrices.

**Results:**

Logistic Regression and Gradient Boosting achieved the highest accuracy (95.11%) and ROC-AUC (0.836), although all models demonstrated poor recall, reflecting challenges in identifying rare stroke cases. Feature importance analysis using the Random Forest model identified age, average glucose level, and BMI as the most influential predictors of stroke, aligning with the Metabolic Syndrome Hypothesis and previous epidemiological findings.

**Discussion:**

These findings underscore both the promise and current limitations of ML in stroke risk prediction and highlight the need for future research leveraging multi-modal datasets and advanced algorithmic strategies to enhance sensitivity and clinical utility.

## 1 Introduction

Stroke is a major global health burden and a leading cause of death and long-term disability, responsible for approximately 10% of global mortality and 5% of all disability-adjusted life years (DALYs) (1). A stroke occurs when blood flow to parts of the brain is disrupted, depriving brain cells of oxygen and nutrients, leading to cell death. It is a medical emergency that requires immediate attention to minimize brain damage and other complications (2). According to the World Health Organization (WHO), around 15 million people worldwide experience a stroke each year, and nearly 6 million die as a result with a death occurring every 4-5 min (3, 4). The two primary types of stroke are ischemic and haemorrhagic, with ischemic strokes caused by blood clots, comprising more than 80% of all cases, especially in high-income regions like the United States (5). Ischemic strokes often result from atherosclerosis or embolic events, with atrial fibrillation (AF) being a major contributing factor (6, 7). Haemorrhagic strokes, although less common, involve ruptured blood vessels and are typically more severe which is caused by bleeding around the brain (8, 9).

The global burden of stroke disproportionately affects low- and middle-income countries (LMICs), where approximately 75% of all stroke cases and 87% of stroke-related deaths occur (10). China, for instance, faces the largest national burden, with 34 million prevalent cases and over 2 million deaths annually (11–13). Stroke also imposes significant economic strain, costing nearly $891 billion globally in 2017 through both direct healthcare and indirect productivity losses (14).

Despite its high burden, stroke is largely preventable. Stroke risk can be reduced through a healthy lifestyle, avoiding smoking and alcohol, maintaining a healthy BMI and glucose levels, and supporting heart and kidney health. Stroke risk increases with prior stroke or transient ischemic attack, myocardial infarction, heart failure, atrial fibrillation, age over 55, hypertension, atherosclerosis, smoking, high cholesterol, diabetes, obesity, sedentary lifestyle, alcohol use, clotting disorders, hormone therapy, and substance abuse (e.g., cocaine, amphetamines) (15, 16). Symptoms often appear suddenly, e.g., unilateral paralysis, speech difficulty, dizziness, or coma, and may occur during sleep (17–19). Diagnosis relies on CT or MRI, with additional tests like carotid or cardiac triplex; treatment depends on stroke type and may involve drugs, surgery, or intensive care, especially within the first 24 h (20, 21). It is estimated that 50% of stroke cases in high-risk populations can be prevented through effective control of modifiable risk factors such as hypertension, diabetes, obesity, smoking, and physical inactivity (22). Traditional risk assessment tools, such as the Framingham Stroke Risk Profile (FSRS) and its revised versions, which rely heavily on clinical risk scores, have long been used to estimate a patient’s 10-year stroke risk based on factors (epidemiological data) like age, blood pressure, smoking, and cardiovascular history (23). However, while these models have been instrumental in guiding treatment decisions, they often assume linear relationships between risk factors and stroke outcomes, potentially oversimplifying and fail to capture complex interactions, limiting their predictive accuracy in diverse real-world populations (24).

Recent advances in artificial intelligence (AI), particularly in machine learning (ML), has emerged as a promising alternative for stroke prediction. Unlike traditional statistical models. ML algorithms can process large, high-dimensional datasets and uncover non-linear patterns between risk factors and stroke occurrence (25, 26). Techniques such as logistic regression, support vector machines (SVM), random forests, gradient boosting, and neural networks have been successfully employed in predictive modelling and demonstrated improved predictive performance in various healthcare applications, including stroke risk assessment and other chronic diseases (15, 27). Nevertheless, important challenges remain, including data imbalance as only few studies have explored this in depth for stroke prediction (28), lack of interpretability, and over-reliance on accuracy as the sole performance metric.

Early prediction is crucial to prevent permanent damage or death. This study addresses these gaps by evaluating and comparing multiple ML models for stroke prediction using a balanced dataset to enhance decision-making in the proposed predictive system. The analysis incorporates clinically relevant metrics, such as ROC-AUC, precision, recall, and specificity, and explores the relative importance of key risk factors, including age, BMI, glucose level, hypertension, smoking status, and work type, aligning with the Metabolic Syndrome Hypothesis and previous epidemiological findings. By integrating clinical insights with data-driven approaches, this research aims to enhance early detection of high-risk individuals and contribute to more effective prevention strategies. By doing so, it seeks to contribute to the ongoing efforts to improve early stroke prediction and inform targeted prevention strategies.

## 2 Literature review

### 2.1 Theoretical frameworks

The theoretical framework guiding this study integrates three complementary models to explain stroke risk and justify the selection of predictive features. (1) The Framingham Risk Score (FRS), serving as a foundational clinical model by quantifying stroke risk based on various factors, (2) the Atherosclerosis and Thrombosis Theory, providing a biomedical rationale, describing how cholesterol buildup leads to arterial narrowing and subsequent thrombotic events, and (3) the Metabolic Syndrome Hypothesis. Each framework offers a distinct perspective on understanding, modelling, and predicting stroke risk, collectively guiding the methodological approach of this research and justify the application of machine learning (ML) to develop a more refined stroke prediction system.

#### 2.1.1 Framingham risk score (FRS)

The Framingham Risk Score (FRS), rooted in the landmark Framingham Heart Study, one of the most influential longitudinal investigations in cardiovascular epidemiology, offers a foundational statistical model for estimating an individual’s 10-year risk of cardiovascular events, including stroke. This model leverages a combination of clinical and demographic variables such as age, systolic blood pressure, diabetes status, smoking behavior, and body mass index (BMI) to assess risk (29). Its integration of both modifiable and non-modifiable risk factors underscores its utility in guiding stroke prevention efforts.

Over time, the FRS has gained widespread validation and has been embedded within clinical guidelines across diverse healthcare systems (23, 30). Despite its prominence, a central critique of the FRS lies in its reliance on additive linear assumptions, which can obscure the complex and often non-linear interactions among risk factors. This shortcoming is particularly pronounced in heterogeneous populations, where traditional models may fail to capture nuanced relationships, precisely the kind of complexity that machine learning (ML) techniques are well-equipped to address.

In this study, the FRS informs the initial selection of predictive features and establishes a reference point for evaluating the performance of ML-based models. Its inclusion is especially pertinent given the well-documented contributions of variables such as BMI, age, hypertension, and diabetes to stroke risk, variables repeatedly validated across traditional and emerging analytical paradigms (31). Furthermore, the FRS highlights the relevance of occupational stress, which recent research has linked to increased cardiovascular burden, particularly in high-demand work environments (32).

As machine learning models are deployed to augment and refine the predictive power of the FRS, this framework serves not only as a benchmark for comparison but also as a theoretical scaffold. It ensures the clinical validity of selected features while facilitating a deeper understanding of how non-linear modelling approaches can enhance stroke prediction. In this way, the FRS provides a vital conceptual bridge between classical epidemiological methods and the evolving capabilities of contemporary data science.

#### 2.1.2 Atherosclerosis and thrombosis theory

The Atherosclerosis and Thrombosis Theory offers a critical biomedical framework for understanding the pathogenesis of stroke, focusing on the physiological mechanisms that underlie vascular occlusion. Central to this theory is atherosclerosis, a progressive condition marked by the accumulation of lipids, inflammatory cells, and fibrous elements within arterial walls. Over time, these deposits form plaques that can rupture, triggering thrombotic events and ultimately leading to ischemic stroke, the most common stroke subtype (33).

This theoretical perspective is particularly salient for informing the inclusion of clinical variables such as cholesterol levels, blood glucose, and body mass index (BMI) in predictive modelling. Both hypercholesterolemia and hyperglycaemia are implicated in endothelial dysfunction and plaque destabilization, while obesity promotes systemic inflammation, a driving force in atherogenesis (34). Empirical evidence further supports this connection, demonstrating that elevated low-density lipoprotein (LDL) levels and increased BMI are strongly associated with heightened stroke risk (Gao et al., 2021).

Importantly, the Atherosclerosis and Thrombosis Theory also intersects with behavioral and lifestyle risk factors. Smoking, for instance, has been shown to exacerbate oxidative stress and inflammation, accelerating arterial damage and thrombogenesis (Ambrose and Barua, 2004). Similarly, sedentary behavior contributes to metabolic dysregulation and vascular dysfunction. These biologically plausible pathways enrich the theoretical underpinnings of stroke risk, extending beyond statistical associations to include mechanistic insight, thus complementing the epidemiological focus of the Framingham Risk Score.

Incorporating this theory into the present study strengthens the clinical rationale behind variable selection and lends credibility to the model’s interpretability. By anchoring machine learning outputs in well-established physiological processes, the study ensures that its findings are not only statistically sound but also biomedically coherent. This integration deepens the explanatory power of the predictive framework, bridging the gap between data-driven insights and clinical relevance.

#### 2.1.3 Metabolic syndrome hypothesis

The Metabolic Syndrome Hypothesis conceptualizes stroke because of interrelated metabolic abnormalities, including central obesity, insulin resistance, hypertension, hyperglycaemia, and dyslipidaemia, that collectively elevate cardiovascular risk (35). Its relevance has intensified amid rising urbanization and lifestyle shifts, especially in developing regions where increased metabolic syndrome prevalence parallels stroke incidence (36). Core components such as elevated BMI and impaired glucose regulation, strongly linked to cerebrovascular outcomes (37), are thus essential for predictive modelling. Within the machine learning context, this framework supports modelling strategies that detect complex, non-linear interactions among co-occurring risk factors, capturing high-risk profiles that conventional models may overlook. Integrating this hypothesis into the present study strengthens both the theoretical grounding and the clinical relevance of selected features, while underscoring the value of ML’s capacity to reflect the multifactorial and synergistic nature of stroke etiology.

Complementing biomedical insights, *psychosocial and behavioral theories* help contextualize the elevated stroke risk among individuals in private or self-employed roles. The Job Strain Model attributes this to chronic occupational stress leading to cardiovascular dysfunction (38). Similarly, *the Biopsychosocial Model* reinforces the study’s findings by framing stroke risk as an interplay between physiological factors and social environments, including work and lifestyle. Broader public health perspectives, such as the Social Determinants of Health (SDOH) framework, justify the inclusion of variables like residence type and employment status, while the Health Belief Model emphasizes the need for targeted education to modify behaviors in high-risk groups (39–42). Collectively, these frameworks support the multifactorial understanding of stroke presented in this study and highlight the value of integrated, data-driven approaches in guiding prevention strategies and policy interventions.

The integration of the Framingham Risk Score, Atherosclerosis Theory, and Metabolic Syndrome Hypothesis grounds the study in clinical, biological, and systemic insights, enhancing the relevance and interpretability of machine learning in stroke prediction.

### 2.2 Risk factors of stroke

Stroke remains part of the leading causes of mortality and disability, cognitive impairment worldwide, with ischemic strokes comprising 65–85% of cases in the Western world and haemorrhagic strokes remaining more disabling (43, 44). In the United States alone, roughly 795,000 stroke events occur annually, with costs exceeding $34 billion and a projected prevalence increase of 3.4 million by 2030 (43, 45). Post-stroke cognitive impairment affects over 70% of survivors and significantly contributes to long-term disability and healthcare burden, though not all cases meet criteria for vascular dementia (46). Dysphagia, prevalent in 40–60% of patients following ischemic stroke, often coexists with malnutrition, both of which are linked to worse recovery, prolonged hospitalization, and increased mortality (47, 48). The burden extends to industrialized nations where stroke accounts for up to 5% of national health expenditure, and incidence continues to rise with aging populations (49–51). In developing countries, a transition from haemorrhagic to ischemic stroke patterns reflects changes in hypertension control and dietary shifts (52). Accurate stroke classification, including etiologic subtypes such as cardioembolic, atherosclerotic, lacunar, and cryptogenic stroke, remains foundational to risk factor analysis (6) (Table 1).

**Table 1:** Recommended targets for modifiable risk factors in stroke prevention (236).

Risk factors	Target/recommendation
Hypertension	<140/90 mm Hg (general); <130/80 mm Hg (with diabetes or renal disease) If normotensive, aim for 10 mm Hg systolic and 5 mm Hg diastolic reduction from baseline
Dyslipidaemia	LDL-C < 70 mg/dL or ≥50% reduction (for atherosclerotic stroke) For non-atherosclerotic stroke, follow ATP III guidelines
Diabetes Mellitus	HbA1c < 7%
Smoking	Complete cessation
Alcohol Consumption	≤2 drinks/day for men; ≤1 drink/day for non-pregnant women
Physical Inactivity	≥30 min of moderate exercise, 1–3 times per week
Diet/Nutrition	Low-fat, low sodium; Mediterranean or DASH diet (with diabetic modifications as needed)
Obesity	BMI between 18.5 and 25 kg/m^2^

This table outlines evidence-based targets for modifiable stroke risk factors, summarizing current guideline recommendations for optimal control of blood pressure, lipid levels, glycaemia, smoking, alcohol consumption, physical activity, diet, and body weight to support primary and secondary stroke prevention.

Stroke arises from a combination of modifiable and non-modifiable risk factors. While age, sex, race, and genetic predisposition are non-modifiable, with stroke incidence doubling every decade after age 55 (53–55), modifiable factors account for the majority of stroke burden globally. Chief among these is hypertension, responsible for over half of all stroke cases worldwide (10, 52, 56). Other significant modifiable risks include diabetes mellitus (57), dyslipidaemia (58), atrial fibrillation (AF) and atrial cardiopathy (53), smoking (59), alcohol use (60), obesity, poor diet, and physical inactivity (61). Notably, cholesterol exhibits a subtype-specific effect (62): elevated total and low HDL cholesterol increase ischemic stroke risk, whereas low total cholesterol correlates with haemorrhagic stroke (55, 63, 64). Statin therapy, despite lowering cholesterol, has been shown to reduce ischemic stroke incidence, although it may slightly elevate haemorrhagic stroke risk in specific populations (58, 65).

Obesity, particularly central adiposity, as measured by waist-to-hip ratio, exacerbates stroke risk via pathways including hypertension, dyslipidaemia, and insulin resistance, with up to 76% of BMI-related stroke risk mediated through these mechanisms (66, 67). Metabolic syndrome, clustering these conditions, nearly doubles the risk of ischemic stroke (35, 68). Inflammatory markers such as high-sensitivity C-reactive protein (hsCRP) and infection burden have also been linked to increased stroke susceptibility (69). Acute infections may serve as short-term stroke triggers, particularly within 14–30 days of onset (70). Environmental exposures, especially fine particulate matter (PM2.5), significantly contribute to ischemic stroke risk and associated mortality (71). Furthermore, genetic loci (specific regions of human DNA) such as 9p21, PITX2, ZFHX3, FOXF2, and GUCY1A3 have been implicated in stroke susceptibility and subtype differentiation (72). This underscores that stroke risk is influenced not only by lifestyle and environment but also by genetics, and incorporating genetic data can enhance the accuracy of subtype-specific stroke prediction.

Given this multifactorial etiology, comprehensive prevention strategies must address clinical, behavioral, environmental, and genetic determinants. Tools like the Framingham Stroke Risk Profile and the ASCVD risk calculator guide clinical decision-making by integrating key variables such as age, blood pressure, cardiovascular history, and ethnicity (29, 68, 73, 74). A multidimensional approach remains essential to effectively reducing the global burden of stroke.

### 2.3 Management, assessment and prevention

Stroke prevention is essential in managing atrial fibrillation (AF), a common cardiac arrhythmia that significantly increases the risk of ischemic stroke by promoting thrombus formation in the atria (75–77), as multiple risk factors, including female sex, elevate AF-related stroke risk. Despite this, women are less likely to receive oral anticoagulation due to perceived bleeding risks and potential underestimation of their thromboembolic risk (78). While advances in acute stroke care and rehabilitation have improved global outcomes (79, 80), post-stroke cognitive decline remains common and contributes substantially to long-term disability.

#### 2.3.1 Stroke prevention—conceptual foundations and levels of intervention

Stroke prevention is a multidimensional process that encompasses a continuum of interventions, primordial, primary, and secondary, designed to mitigate cerebrovascular risk across the lifespan. At the foundational level, primordial prevention aims to eliminate the emergence of risk factors within populations through structural, educational, and behavioral health strategies (30, 81). These include reducing tobacco use, encouraging healthy dietary habits, increasing physical activity, and addressing socioeconomic determinants of health. Primary prevention targets individuals with identifiable risk factors such as hypertension, diabetes, and dyslipidaemias but without prior cerebrovascular events, aiming to prevent the initial occurrence of stroke. Meanwhile, secondary prevention is focused on patients who have experienced a stroke or transient ischemic attack (TIA), utilizing pharmacologic and non-pharmacologic strategies to reduce the risk of recurrence (82). These levels are interconnected; effective population-level prevention supports individual risk modification, and tailored clinical management reinforces broader public health goals.

#### 2.3.2 Assessment and risk stratification—from population metrics to individualized profiles

Accurate risk assessment is a prerequisite for effective stroke prevention and management. This involves both population-based tools and individual clinical evaluations. For general cardiovascular risk estimation, tools such as the Framingham Stroke Risk Profile (83) and the ASCVD Risk Estimator (73) are widely validated. These integrate age, sex, systolic blood pressure, diabetes, smoking status, cholesterol levels, and the presence of cardiovascular comorbidities. In specific populations, particularly those with atrial fibrillation (AF), risk stratification models such as the CHA₂DS₂-VASc score are used to determine thromboembolic risk and guide anticoagulation therapy (84). Clinical evaluation must also include routine blood pressure monitoring, lipid profiles, glucose/HbA1c testing, and anthropometric measurements such as body mass index (BMI) and waist-to-hip ratio. The dynamic nature of stroke risk, affected by aging, comorbid conditions, and medication adherence, necessitates periodic reassessment to refine preventive strategies over time.

#### 2.3.3 Management of modifiable risk factors—lifestyle, pharmacology, and multimodal integration

The effective management of modifiable stroke risk factors remains the most actionable and evidence-based component of stroke prevention, particularly in secondary prevention settings. Hypertension, the most critical determinant of stroke risk, must be addressed aggressively; even modest reductions in blood pressure (e.g., 5–6 mm Hg diastolic) have been shown to reduce stroke incidence by up to 40% (85). Similarly, while strict glycaemic control has not consistently demonstrated reductions in macrovascular events in diabetic patients, its benefits for microvascular outcomes and overall cardiovascular risk justify ongoing treatment (86). Lipid-lowering therapy, particularly with statins, has a strong evidence base, showing a 25–30% reduction in stroke risk among high-risk populations, including those with previous cardiovascular disease (87). Lifestyle interventions, smoking cessation, alcohol moderation, structured physical activity, and dietary changes, are equally critical. Smoking cessation alone halves stroke risk within 5 years (88), quitting smoking after a stroke or TIA markedly lowers the risk of recurrence and mortality compared to continued smoking (89), while moderate-intensity exercise is associated with a significantly reduced risk of ischemic stroke (90). These interventions must be sustained and supported by structured counselling, patient education, and multidisciplinary care models.

#### 2.3.4 Pharmacological and procedural strategies: evidence-based therapeutics and risk–benefit considerations

Beyond lifestyle and medical risk factor control, targeted pharmacological interventions play a pivotal role in reducing stroke recurrence, particularly in high-risk patients. Antiplatelet agents, including aspirin and clopidogrel, are central to secondary prevention in patients with non-cardioembolic ischemic stroke or TIA. Meta-analyses confirm that aspirin, in doses of 75–325 mg daily, reduces the risk of recurrent stroke by approximately 25% (91). Clopidogrel provides a modest additional benefit and is particularly recommended in aspirin-intolerant individuals (92). In cardioembolic stroke, especially due to atrial fibrillation, anticoagulant therapy is essential. Trials such as EAFT and subsequent analyses show that warfarin reduces stroke risk by 68–70% (84, 93). More recently, direct oral anticoagulants (DOACs) such as apixaban and rivaroxaban have demonstrated superior safety profiles with comparable or greater efficacy (94). In certain cases, procedural interventions, such as carotid endarterectomy (CEA) in asymptomatic patients with significant stenosis, may be appropriate, though only when operative risk is low and life expectancy exceeds 5 years (95). Ultimately, prevention requires an integrated framework of clinical vigilance, evidence-guided decision-making, and patient-centred care.

Despite the substantial advances in traditional stroke prevention and management, including pharmacological therapies, lifestyle interventions, and risk stratification tools such as the Framingham Stroke Risk Profile and CHA₂DS₂-VASc, these approaches remain limited in their ability to capture the complex, dynamic, and multifactorial nature of stroke risk. Traditional models often rely on linear assumptions and static variables, which may inadequately reflect individual patient trajectories, temporal changes in physiology, and nuanced interactions among clinical, genetic, behavioral, and environmental factors (96). Moreover, they underperform in diverse populations and offer limited personalization. In this context, machine learning (ML) presents a transformative opportunity to enhance stroke prediction, diagnosis, and treatment by leveraging large-scale, heterogeneous datasets, such as electronic health records, imaging, genomics, and wearable sensor data, to model non-linear relationships and discover latent risk patterns (97). By continuously learning from new data and adapting to individual-level variation, ML algorithms offer a scalable, data-driven complement to traditional methods, potentially improving accuracy, equity, and clinical decision-making across the stroke care continuum.

### 2.4 AI-based risk modelling in healthcare

Although traditional stroke risk models, such as the Framingham Stroke Risk Profile (FSRP) and Cox proportional hazards regression have long served as the foundation for risk prediction and prevention strategies, their effectiveness is increasingly being questioned. These models, while clinically useful, are inherently limited by their reliance on a small number of predetermined risk factors, linear associations, and static input parameters that fail to capture the complexity of patient-specific risk in real-world settings (98, 99). Furthermore, these tools often perform inconsistently across populations due to their reduced adaptability to diverse demographic, clinical, and physiological profiles. For example, the FSRP has been shown to underestimate stroke risk in Chinese cohorts, particularly among high-risk men and older adults, suggesting significant calibration limitations (100). In contrast, machine learning (ML) models have emerged as a powerful alternative capable of addressing these limitations by leveraging non-linear algorithms, high-dimensional data, and adaptive learning structures to enhance predictive accuracy. ML approaches such as random survival forests (RSF), gradient-boosted trees (GBT), support vector machines (SVM), and deep neural networks (DNNs) have consistently demonstrated superior performance in predicting stroke and cardiovascular events across varied clinical contexts (101, 102).

Risk prediction models are fundamental tools in clinical decision-making, traditionally built using statistical methods like Cox regression or logistic models, as seen in well-established scores such as FSRP and CHA₂DS₂-VASc. These models offer interpretability and simplicity but are often constrained by limited feature capacity and linear assumptions, reducing their predictive accuracy (98). In contrast, ML-based approaches enable modelling of complex, non-linear interactions and higher-order associations among diverse predictors, offering gains in both discrimination and calibration (98). Importantly, ML can integrate large-scale, multimodal data, including demographic, clinical, imaging, and genomic variables, allowing it to personalize risk predictions and uncover novel determinants of stroke (98). However, the adoption of ML in clinical settings is not without challenges. A major concern is the interpretability of ML algorithms, especially ensemble and neural network models, which are often viewed as “black boxes.” In response, explainable AI tools like Shapley Additive Explanations (SHAP) have been developed to quantify feature importance and enhance model transparency (103, 104). Additionally, adherence to reporting standards such as TRIPOD is essential to ensure model validity and reproducibility. Ultimately, while ML offers a theoretical and empirical advantage over conventional models, its utility in stroke prediction hinges on balancing predictive performance with transparency, fairness, and clinical applicability.

### 2.5 Machine learning methods for stroke risk prediction

Machine learning (ML) is a branch of artificial intelligence that enables computer systems to learn patterns from data and make data-driven predictions or decisions without being explicitly programmed. A machine learning model improves its performance at task T (e.g., stroke prediction), as measured by performance P (e.g., accuracy or AUROC), through experience E (e.g., learning from patient records) (105). ML is broadly categorized into supervised learning, unsupervised learning, reinforcement learning, and deep learning. In stroke risk prediction*, supervised learning*, where models are trained on labelled datasets to predict outcomes such as stroke occurrence, is most widely used. Common algorithms include logistic regression, support vector machines (SVM), random forests (RF), and gradient-boosted trees (GBT), known for their interpretability and effectiveness (99). *Unsupervised learning*, which identifies hidden patterns in unlabelled data, is less commonly applied but useful in phenotyping stroke subtypes or clustering patient profiles (106). *Reinforcement learning*, which optimizes decisions through trial-and-error interactions with an environment, is emerging in clinical decision support but remains underexplored in stroke care. *Deep learning*, a subset of ML using layered neural networks, excels in analyzing complex, high-dimensional data such as imaging and ECGs, outperforming traditional models in some predictive tasks (101). As ML continues to evolve, its integration into stroke prediction promises greater precision, personalization, and scalability compared to conventional risk scoring methods.

Various supervised machine learning techniques have been used to predict stroke occurrence or outcomes based on patient data such as demographics, medical history, vital signs, and lab results. Common algorithms include both classical models and deep learning approaches:

#### 2.5.1 Logistic regression (LR)

LR is widely used in medical research for modelling binary outcomes due to its ability to quantify relationships between outcome variables and various types of predictors, including nominal, ordinal, interval, and ratio-level data (107, 108). Popularly used for binary classification, it estimates the probability of an outcome belonging to a specific class, despite its name suggesting a regression technique (109). It uses the sigmoid (logistic) function to compute probabilities and applies a decision boundary to classify data points (110). Its appeal lies in its interpretability and straightforward implementation. However, LR is limited to modelling linear associations and is susceptible to issues such as multicollinearity and variance inflation, which can reduce predictive accuracy (111, 112). These limitations restrict its effectiveness in capturing the complex, non-linear relationships often present in acute stroke prognosis (108).

The mathematical equation for logistic regression shown in Equation 1 is based on the logistic (sigmoid) function and models the probability that a given input x belongs to a particular class (typically class 1 in binary classification):


(1) $$P_{i}=\frac{1}{1+e^{-\sum_{j=0}^{M}\beta_{j}x_{i}j}}$$

Where,

i = 1…N (number of observations).

j = 1…M (number of individual variables).

𝑝𝑖 = predicted probability of a ‘1’ at observation i.

𝛽𝑗= Regression Coefficient.

𝑥𝑖𝑗= The j-th variable at observation i.

Although LR is limited in capturing non-linear interactions, recent studies show it can perform competitively with proper data preprocessing. For instance, one study achieved approximately 86% accuracy using LR by applying data imputation, outlier removal, and the Synthetic Minority Oversampling Technique (SMOTE) to correct class imbalance (113). The model included key predictors such as blood pressure, body mass index (BMI), cardiac history, age, smoking status, glucose levels, and prior stroke events. These findings reinforce that, while ML methods may better capture complex interactions, LR remains a practical, transparent, and effective tool for stroke risk assessment when supported by rigorous data engineering.

#### 2.5.2 Support vector machines (SVM)

Support Vector Machines (SVM) are a robust machine learning method grounded in statistical learning theory, capable of addressing both classification and regression tasks (114). SVMs classify data by identifying an optimal hyperplane that separates data points, represented as n-dimensional vectors, into distinct classes. Through kernel functions, SVMs can effectively manage both linear and non-linear datasets, making them particularly useful for complex medical data (115). In stroke prediction, SVMs have been extensively applied, especially in earlier studies, due to their ability to handle high-dimensional feature spaces and model non-linear relationships. A systematic review by (116) found that SVM was the best-performing model in 10 out of 39 stroke-related studies conducted between 2007 and 2019. These studies demonstrated SVM’s effectiveness in both stroke diagnosis and outcome prediction using clinical and imaging data. While more recent models such as ensemble methods often outperform SVMs in terms of raw predictive accuracy, SVM continues to serve as a strong benchmark model. For instance, Vu et al. (117) included SVM in their comparative analysis of classifiers for stroke incidence prediction in the Suita cohort, where SVM maintained competitive accuracy. Moreover, SVMs are frequently integrated into feature selection workflows due to their capacity to identify support vectors, data points that are most critical to the decision boundary. However, SVM performance is highly sensitive to hyperparameter tuning and data imbalance. As such, strategies like feature scaling, cost-sensitive training, and the use of SMOTE are commonly employed to enhance its utility in stroke datasets (103).

The following terms in Equation 2 can be used to define a support vector classifier:


(2) $$f(x)=\mathrm{\beta ο}+\sum_{\mathrm{i\epsilon S}}a_{i}k(x_{1},\mathrm{xi})$$

Where, β0 = bias, S = set of observations, *α* = model parameters that must be learned.

Support Vector Machines (SVM) handle multiclass classification using one-vs-all or one-vs-one strategies and are widely recognized for their effectiveness in supervised learning tasks, including classification and outlier regression (118).

#### 2.5.3 K-Nearest Neighbours (KNN)

KNN is a simple, non-parametric, instance-based learning algorithm often used in stroke prediction as a baseline or supporting model. Unlike models that learn a function from the training data, KNN classifies new observations by analysing the majority class among their k nearest neighbors in an n-dimensional feature space, based on similarity metrics such as Euclidean distance (119–121). While its simplicity and intuitiveness make KNN widely accessible, it can perform poorly in high-dimensional or imbalanced datasets due to sensitivity to irrelevant features and noise. Consequently, KNN is rarely the top-performing model in stroke risk prediction but is commonly included in comparative analyses or ensemble methods. For example, (122) used KNN as the final estimator in a stacking ensemble alongside random forest and decision trees, achieving high predictive accuracy (98.6%). In such settings, KNN benefits from the aggregated insights of stronger base learners. Furthermore, techniques like principal component analysis (PCA) can enhance KNN’s performance by reducing feature dimensionality and improving neighbour similarity, mitigating the “curse of dimensionality” often encountered in clinical datasets.

The KNN mathematical equation is described by Equation 3:


(3) $$d_{\text{Euclidean}}=\sqrt{\sum_{i=1}^{n}({x_{i}}^{2}-{y_{i}}^{2})}$$

The model’s performance will be presented in the results section of this study.

#### 2.5.4 Decision trees (DTs)

DTs are a supervised learning method that classifies a target variable by recursively learning simple decision rules from input features (123). These rules split variables based on impurity measures (e.g., Gini index, entropy) until a stopping criterion is met (110). Visually, a decision tree resembles an inverted tree, with the root node at the top and branches representing feature-based splits. Decision trees are widely used in stroke prediction for their interpretability, ability to model non-linear relationships, and capacity to handle mixed data types.

Among tree-based models, Random Forests (RF), ensembles of multiple decision trees, have become a dominant approach in stroke risk prediction. RF models improve accuracy by aggregating predictions from many de-correlated trees, reducing overfitting and automatically modelling feature interactions. A systematic review by Asadi et al. (27) found RF to be the top-performing algorithm in 25% of stroke-related ML studies between 2019 and 2023. Studies by Choudhury et al. (2023) and Vu et al. (117) similarly reported RF outperforming models like logistic regression, SVM, and even deep neural networks. Additionally, RF offers insights into feature importance, as demonstrated in (104) explainable-AI study, where SHAP analysis highlighted age, triglyceride levels, and aphasia as key predictors. RF’s explainability and predictive strength make it especially valuable in clinical settings. *Gradient boosting* variants like XGBoost and LightGBM have also shown strong results, with XGBoost ranking second to RF in multiple studies (104). Collectively, tree-based ensembles, particularly Random Forest, remain a cornerstone of modern stroke prediction research due to their robustness, accuracy, and interpretability.

#### 2.5.5 Naïve Bayes (NB)

NB is a probabilistic, supervised learning classifier that assumes conditional independence among features and has been explored in stroke risk prediction for its simplicity and efficiency. Despite its strong independence assumption, NB can perform surprisingly well when features are informative and relatively uncorrelated. In structured health datasets, NB has occasionally outperformed more complex models; for example, a study reviewed in Nature reported NB achieving the highest accuracy (~82%) for early stroke identification, likely due to dataset characteristics and predictor strength (124). Although NB is rarely the leading method in the current era of deep learning, it remains a valuable baseline in comparative studies and is often incorporated into ensemble frameworks (e.g., bagging or voting) to enhance predictive robustness. Its low computational cost and interpretability make it particularly useful in smaller or cleaner clinical datasets (124, 125).

#### 2.5.6 Deep learning

Neural Networks has gained increasing attention in stroke risk prediction, particularly with the availability of large and complex datasets. Models such as Multilayer Perceptron (MLP), Convolutional Neural Networks (CNNs), and Recurrent Neural Networks (RNNs), including Long Short-Term Memory (LSTM) networks, have been applied to both structured tabular data and heterogeneous sources like clinical records, imaging, and time-series signals. A major strength of deep learning (DL) lies in its capacity to automatically learn feature representations, removing the need for manual feature engineering. Moulaei et al. (126) highlighted DL’s growing role in stroke management, particularly in acute stroke diagnosis and imaging-based detection, due to its speed and effectiveness (104, 127). In stroke risk prediction using clinical data, the debate has centered on whether DL can consistently outperform well-optimized traditional machine learning (ML) models. Until recently, most studies evaluated DL and ML separately, limiting direct comparison (128–131). Addressing this gap, (104) compared eight models (four ML and four DL) on the same dataset, finding that while Random Forest (RF) had the best overall performance, DL models as a group outperformed other ML methods in both accuracy and sensitivity (104). Notably, LSTM networks achieved a sensitivity of 96.15%, and the Feedforward Neural Network (FNN) variant showed the highest specificity and F1-score among DL models (104). Another study cited by Heo et al. (132) reported ~94% accuracy using LSTM on EEG data for stroke prediction, demonstrating DL’s potential with alternative modalities. CNNs, though traditionally used for image analysis, have been adapted for tabular data by encoding clinical features as “image-like” inputs, capturing local feature interactions effectively (133, 134). Despite these advances, DL models are often criticized for their black-box nature, which limits clinical interpretability. To address this, explainability techniques such as SHAP and layer-wise relevance propagation are increasingly applied to interpret predictions (104, 135). Ultimately, DL is emerging as a high-performing approach in stroke risk modelling, particularly when rich and diverse datasets are available.

### 2.6 Other related studies and hybrid approaches

In addition to individual machine learning (ML) algorithms, hybrid and ensemble strategies have been increasingly explored in stroke prediction to enhance accuracy and robustness. Stacking ensembles, where multiple base learners are combined using a meta-learner, have demonstrated state-of-the-art performance by leveraging the strengths of diverse models. A study by Alam et al. (2024) reported 98.6% accuracy using a stacked ensemble comprising Random Forest, Decision Tree, and K-Nearest Neighbours (KNN) classifiers on a public stroke dataset (BMC Bioinformatics). Such hybrid models can counterbalance individual algorithm biases, improving predictive equity, particularly in imbalanced data scenarios (136). Boosting algorithms like XGBoost and LightGBM also feature prominently in ensemble configurations, offering high performance through iterative learning.

Unsupervised learning methods, though less common, are increasingly used to complement supervised models. For instance, Vu et al. (117) applied k-prototypes clustering to segment a cohort of 7,389 individuals into risk subgroups, enabling tailored predictions by subsequent supervised models such as RF, SVM, and XGBoost (98). This two-step approach improved risk stratification and model personalization. Hybrid methods also include novel integrations, such as combining ML with computational fluid dynamics to simulate stroke-related arterial flow, which may enrich mechanistic understanding alongside predictive modelling (98).

Emerging algorithms like Minimal Genetic Folding (MGF) have also been tested (137). One study achieved 83% accuracy with an MGF-based model, slightly outperforming traditional classifiers, though it required data oversampling and remains experimental (137). Comprehensive reviews consistently show ensemble and hybrid models outperforming standalone methods, particularly in complex or imbalanced clinical datasets, highlighting their growing significance in stroke risk modelling (103).

#### 2.6.1 Recent contributions

In the past 5 years, stroke risk prediction has witnessed major advancements through the application of machine learning (ML), with significant improvements in predictive accuracy and *methodological sophistication* (27). Recent studies have reported model accuracies frequently exceeding 90%, compared to earlier averages around 80%, largely due to enhanced data preprocessing techniques (e.g., SMOTE, SMOTE-ENN), inclusion of broader clinical variables, and the adoption of ensemble and hybrid methods such as stacking (103, 104, 122, 124, 138). Moulaei et al. (104) demonstrated that while Random Forest (RF) remained the top overall performer, deep learning model, particularly LSTMs, achieved superior sensitivity (104). These head-to-head comparisons have provided greater clarity on model selection under standardized conditions. Further, the use of real-time prediction with wearable devices (139), and explorations of transfer and multi-task learning, reflect the field’s shift toward dynamic, personalized risk assessment.

Alongside performance gains, recent ML studies have deepened clinical insight by identifying both established and *novel stroke risk factors*. Tools such as SHAP have consistently ranked age, hypertension, diabetes, smoking, and atrial fibrillation as primary contributors (117, 124), while also revealing additional predictors like fructosamine, haemoglobin, calcium, skinfold thickness, triglyceride levels, and aphasia, many of which are absent from traditional risk scores (104). This dual role of ML, as a predictive and exploratory tool, has opened new avenues for research and refined risk stratification strategies. Moreover, ML has expanded into specific *clinical subdomains* such as post-stroke mortality, functional recovery, haemorrhagic complications, cognitive impairment and perioperative stroke risk (122, 132, 140). These applications demonstrate how machine learning methods not only improve general population-level predictions but also support targeted, domain-specific clinical decisions. The growing emphasis on model explainability further strengthens the clinical utility of ML by enhancing interpretability, trust, and translational potential.

In a study by Shoily et al. (141), four ML algorithms, Naive Bayes, J48, K-nearest neighbour, and Random Forest, were applied, with J48, KNN, and Random Forest achieving an impressive 99.8% accuracy, while Naive Bayes reached 85.6%. A social media-based approach in (142) used spectral clustering on tweets, applying Naive Bayes, support vector machine, and probabilistic neural networks (PNN), with PNN performing best at 89.90% accuracy. In (143), a comparative study of nine classifiers found that a boosting model with decision trees achieved the highest recall (99.94%), and Random Forest delivered the best precision (97.33%). The widely used Kaggle stroke dataset was employed in (138) who achieved 82% accuracy using various classifiers. In (144), data from Sugam Multispecialty Hospital, India, showed that ensemble methods and support vector machines provided 91% accuracy, while artificial neural networks trained with stochastic gradient descent exceeded 95%. An EHR-based analysis in Nwosu et al. (145) evaluated neural networks, decision trees, and Random Forests, with accuracies of 75.02, 74.31, and 74.53%, respectively. Lee et al. (129) explored ML for analysing diffusion-weighted imaging (DWI) and fluid-attenuated inversion recovery (FLAIR) images within 24 h of symptom onset, using logistic regression, support vector machines, and Random Forests to estimate stroke onset time, demonstrating comparable or superior performance to human interpretation based on sensitivity and specificity.

Govindarajan et al. achieved 95% accuracy using ANN and SGD on data from 507 patients. Amini et al. used C4.5 and KNN on 807 subjects with 50 risk factors, attaining 95 and 94% accuracy, respectively (146, 147). Cheng et al. (148) employed two ANN models for ischemic stroke prognosis with up to 95% precision. Cheon et al. (128) used a deep neural network and PCA on 15,099 patients, reaching an AUC of 83%. Singh & Choudhary (149) applied decision trees, PCA, and neural networks on the CHS dataset, achieving 97% accuracy. Chin et al. (150) developed a CNN-based system for early ischemic stroke detection using 256 images, achieving 90% accuracy. Sung et al. (151) used linear regression and data mining on 3,577 cases to build a stroke severity index, with KNN yielding the best results. Monteiro et al. (152) predicted ischemic stroke outcomes three months post-admission with an AUC above 90%. Kansadub et al. (153) found Decision Tree and Naive Bayes most accurate among tested models. Adam et al. (154) compared KNN and Decision Tree for classifying ischemic stroke, favoring the latter for clinical use.

Despite significant advancements in machine learning (ML) for stroke risk prediction, several challenges remain unresolved in the literature. Chief among these is class imbalance, limited external validation, and the trade-off between predictive performance and interpretability. Stroke datasets often exhibit a skewed distribution, with relatively few positive cases, leading to biased models if left uncorrected. While recent studies have adopted oversampling strategies, not all rigorously evaluate their impact. The complexity of deep learning models has also raised concerns around explainability and clinical trust, particularly in high-stakes applications like stroke prevention (27, 124).

In response, this study addresses several of these concerns through a multi-faceted approach. To mitigate class imbalance, *Random Over-Sampling* was employed to increase the representation of stroke cases during model training. This straightforward yet effective technique allowed for balanced learning without introducing synthetic data. This approach aligns with best practices in interpretable ML, emphasizing domain-informed model design. The model ensemble incorporated interpretable algorithms, such as logistic regression and KNN, alongside more complex classifiers like random forests, SVM and gradient boosting, allowing for both performance and transparency. This work also addresses the often-overlooked inclusion of lifestyle-related variables in stroke prediction models. By incorporating features like smoking history and BMI, elements typically missing in models relying solely on clinical variables, our analysis enhances the model’s relevance to real-world prevention strategies. Feature importance was evaluated using built-in model metrics, offering insights into key risk factors without relying on black-box techniques. Importantly, our feature selection was guided by a theoretical framework rooted in clinical and epidemiological evidence, ensuring variables like age, blood pressure, BMI, diabetes, and smoking were chosen for their known relevance to stroke risk. By including clinical, demographic, and behavioral variables, and benchmarking performance across diverse models, this work contributes to the development of robust, interpretable, and clinically meaningful stroke prediction tools (27, 103).

To address these gaps, this study investigates:

1. How accurately can supervised ML algorithms predict stroke occurrence using demographic, clinical, and lifestyle data?
2. Which features most significantly influence stroke predictions, and how do they align with established clinical risk factors?

## 3 Methods

This study employed a structured machine learning approach specifically designed to address our primary research objective: developing an accurate and clinically interpretable stroke prediction model. Our methodological framework was constructed to balance predictive performance with clinical utility, addressing the critical need for early stroke risk identification in diverse patient populations based on demographic, clinical, and lifestyle factors, while explicitly addressing methodological challenges such as class imbalance, interpretability, and generalizability. This study adopts a quantitative research design utilizing machine learning (ML) techniques to develop predictive models for stroke risk classification. The methodology encompasses several phases: data acquisition and preprocessing, exploratory data analysis, feature selection, model development using five supervised learning algorithms, evaluation using appropriate performance metrics, and strategies for addressing data imbalance. Leveraging a structured machine learning pipeline, the study followed a systematic process encompassing problem definition, data preprocessing, model development, and evaluation. Supervised learning was adopted due to the availability of labelled outcome data (155), thus enabling direct mapping between patient characteristics and stroke occurrence. This aligns with our secondary objective of identifying the most significant predictors of stroke risk, which required algorithms capable of feature importance quantification. Furthermore, our emphasis on cross-validation and performance metrics evaluation directly supports our aim to develop models that maintain reliability across different patient subgroups, addressing the clinical challenge of generalizability in stroke prediction. To ensure data quality and machine-readability, the dataset was cleaned, encoded, and refined by selecting clinically relevant features (156, 157). We employed a 4:1 train-test split and this is particularly appropriate for our stroke prediction task as it provides enough training data to capture the complex relationships between clinical variables and stroke outcomes, while the 20% test portion remains large enough to include adequate representation of the minority stroke class despite its rarity in the dataset. This split ratio has demonstrated empirical validity in similar clinical prediction tasks (158) and, when combined with our five-fold cross-validation strategy, provides a comprehensive framework for assessing model generalizability. Techniques such as regularization and cross-validation were used to reduce overfitting and enhance model robustness (159, 160).

### 3.1 Data information and preprocessing

The dataset utilized in this study was obtained from Kaggle’s publicly available *Stroke Prediction Dataset* (Soriano, 2021), comprising 5,110 individual records with 11 features spanning demographic (e.g., age, gender), clinical (e.g., hypertension, heart disease, glucose level), and lifestyle factors (e.g., smoking status, work type, residence type). Table A1 shows in appendix the data description. Initial exploratory data analysis guided the identification of missing values, data types, and class distribution. Notably, stroke cases accounted for only 4.9% of the total, highlighting a significant class imbalance (0 = 4,861; 1 = 249). 201 missing BMI values were imputed using the mean, a standard approach in clinical data preprocessing when missingness is low and assumed to be random. This method maintains dataset size and model stability without adding synthetic variability, consistent with best practices in medical ML modelling (161, 162).

To address this imbalance, Random Over-Sampling was applied to the training folds, increasing the representation of the minority class. This technique was preferred over synthetic sampling methods like SMOTE to preserve the natural feature distribution, retain data interpretability, and minimize synthetic artifacts (163, 164). To prevent overfitting and information leakage, stratified five-fold cross-validation was implemented post-resampling, ensuring class proportions were preserved in each fold and evaluation was conducted on unseen data (165). Confusion matrices were also generated for each model to visualize classification performance in terms of true/false positives and negatives.

For preprocessing, categorical variables were label- or one-hot encoded, and continuous variables were normalized using Min-Max scaling to a [0,1] range. This standardization step ensured that features were on a uniform scale, which is essential for algorithms sensitive to feature magnitude, such as SVM and KNN (166). The BMI variable had 201 missing observations, which were imputed using the column mean, an approach suitable under the assumption of data missing at random (MAR) and normally distributed values. While mean imputation can reduce variability, it maintains dataset size and is commonly applied in clinical ML contexts (167).

Feature selection was guided by both domain knowledge and data-driven techniques. Variables such as age, BMI, diabetes status, smoking behavior, and blood pressure were included based on their established roles in stroke pathophysiology (29, 35, 83, 168). Additionally, feature importance scores from ensemble models like Random Forest were used to validate and rank predictive variables such as average glucose level, age, BMI and hypertension (169, 170). This comprehensive preprocessing pipeline ensured that the dataset was analytically sound and suitable for robust model development and evaluation.

### 3.2 Machine learning model development

To develop and evaluate predictive models for stroke classification, this study employed five supervised machine learning algorithms: logistic regression (LR), random forest (RF), gradient boosting machine (GBM), support vector machine (SVM), and k-nearest neighbours (KNN). These models were selected for their proven efficacy in clinical risk prediction and their complementary strengths across interpretability, flexibility, and performance (140). *Logistic regression* was chosen as a baseline due to its widespread use in epidemiological modelling and its interpretability in quantifying the relationship between independent variables and stroke risk (29). To address the limitations of linear models, tree-based ensemble methods such as *random forest* and *gradient boosting* were introduced. Random forest, through its bootstrap aggregation of decision trees, mitigates overfitting and captures complex feature interactions, while gradient boosting sequentially improves predictions by minimizing error in a stage-wise fashion, making it particularly effective in structured health data (171). *Support vector machines* were included for their capacity to handle high-dimensional feature spaces and non-linear relationships via kernel functions, a valuable trait given the multifactorial nature of stroke risk (118). *K-nearest neighbours*, though less complex, provided a non-parametric benchmark model that classifies observations based on feature similarity; its inclusion offered insights into the performance of distance-based methods under class imbalance. All models were implemented using Scikit-learn and related libraries in Python and trained on a balanced dataset using an 80/20 train-test split. Hyperparameters were optimized through five-fold stratified cross-validation, with particular attention to maximizing recall and ROC-AUC, metrics that are clinically significant in minimizing false negatives and improving stroke risk identification (164, 172).

### 3.3 Model evaluation

Model performance was evaluated using *5-fold cross-validation*, and assessed using a suite of metrics: *accuracy, F1-score, specificity, AUROC*, and *confusion matrix*, which collectively offer a robust and balanced assessment of classifier effectiveness, especially under imbalanced class distributions (173).

#### 3.3.1 Accuracy

The accuracy, the overall correctness of the classifier, measures the proportion of correctly predicted instances (both positives and negatives) among all observations. It is calculated as shown in Equation 4:


(4) $$\text{Accuracy}=\frac{\mathrm{TP}+\mathrm{TN}}{\mathrm{TP}+\mathrm{TN}+\mathrm{FP}+\mathrm{FN}}$$

where *TP* = true positives, *TN* = true negatives, *FP* = false positives, and *FN* = false negatives.

While intuitive, accuracy can be misleading in imbalanced datasets (174).

#### 3.3.2 F1-score

The F1-score is the harmonic mean of precision and recall, providing a balanced metric in the presence of class imbalance. It is computed as shown in Equation 5:


(5) $$F-\text{score}=2*\frac{\text{Precision}*\text{Recall}}{\text{Precision}+\text{Recall}}$$

Where Precision (the portion of true stroke prediction among all positive predictions) is shown in Equation 6:


(6) $$\text{Precsion}=\frac{\mathrm{TP}}{\mathrm{TP}+\mathrm{FP}}$$

Recall (sensitivity), the model’s ability to identify actual stroke cases, crucial in medical diagnosis is shown in Equation 7:


(7) $$\text{Sensitivity}/\text{Recall}=\frac{\mathrm{TP}}{\mathrm{TP}+\mathrm{FN}}$$

This measure is especially useful when false negatives and false positives carry similar costs (156).

#### 3.3.3 Specificity

Specificity (true negative rate) quantifies the proportion of actual negatives correctly classified as described in Equation 8:


(8) $$\text{Specificity}=\frac{\mathrm{TN}}{\mathrm{TN}+\mathrm{FP}}$$

It is essential in medical diagnostics to avoid false positives as it carries significant consequences (175).

#### 3.3.4 AUROC (area under the receiver operating characteristic curve)

AUROC evaluates a model’s ability to distinguish between classes at various thresholds. This identifies the actual stroke cases, crucial in medical diagnosis. Values range from 0.5 (no discrimination) to 1 (perfect discrimination), summarizing model performance across all classification thresholds (176).

#### 3.3.5 Confusion matrix

A confusion matrix is a performance summary table showing true positives (TP), true negatives (TN), false positives (FP), and false negatives (FN). It supports the computation of key classification metrics and helps evaluate specific types of errors (177).

These metrics were selected based on their relevance to clinical risk classification, where *false negatives* (undetected stroke risk) are more critical than false positives.

### 3.4 Ethical consideration

This study employed a publicly available, anonymized dataset for stroke prediction, with no involvement of human participants or access to personally identifiable information. The dataset adheres to open data licensing standards and was used solely for academic research (178). Despite its secondary nature, ethical integrity was maintained throughout. All data handling, preprocessing, transformation, and resampling was conducted in accordance with data protection principles, such as those outlined in the General Data Protection Regulation (GDPR) for anonymized datasets (179).

The development of machine learning models was informed by ethical AI practices to minimize algorithmic bias and enhance transparency, especially given the clinical implications of stroke prediction. Techniques such as dataset balancing, fairness-aware evaluation, and interpretability strategies were employed to mitigate potential disparities and promote accountable outcomes (180). This research aligns with broader ethical guidelines, including the Declaration of Helsinki (181) and principles for responsible AI in healthcare (182), emphasizing respect for data subjects, beneficence, and algorithmic accountability.

## 4 Results

**Figure 1:**
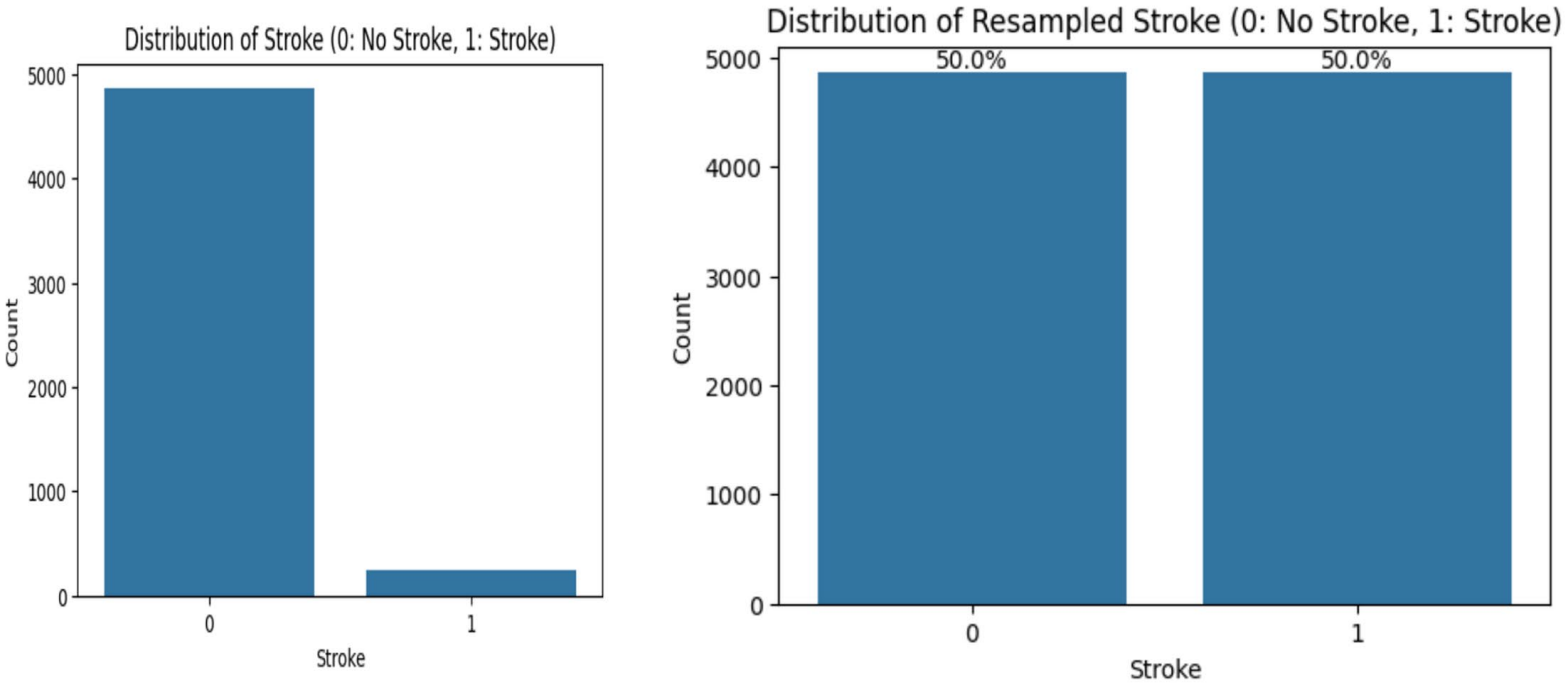
Imbalanced and balanced datasets.

Initial exploration of the dataset revealed a significant class imbalance: only 249 of 5,110 observations (approximately 4.9%) indicated stroke. After applying random oversampling, the distribution was equalized to a 50:50 ratio between stroke and non-stroke classes (Figure 1). This adjustment was critical to enable fairer training conditions and improve sensitivity, especially given the tendency of classifiers to favor the majority class in imbalanced datasets.

### 4.1 Models performance

**Figure 2:**
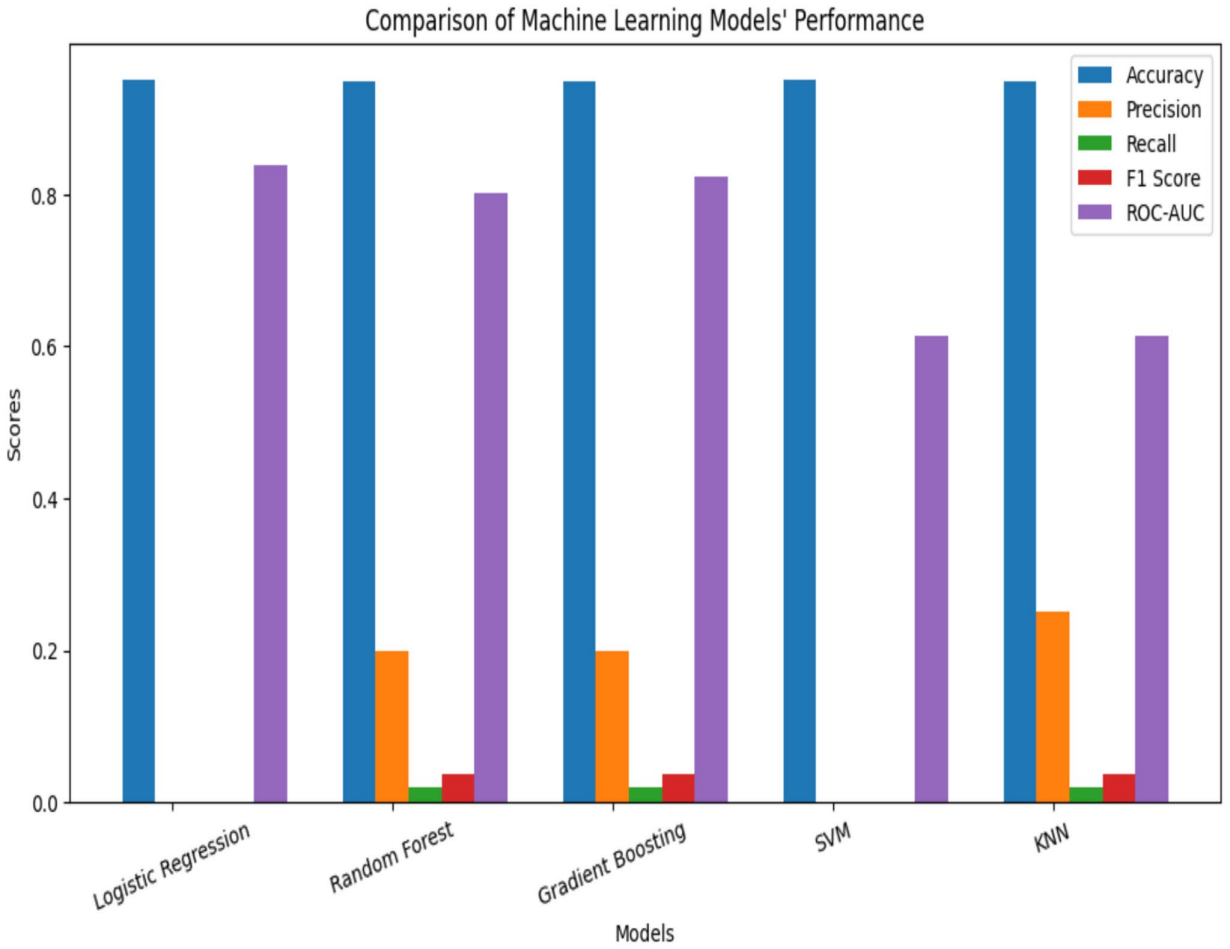
Model accuracy performance.

**Figure 3:**
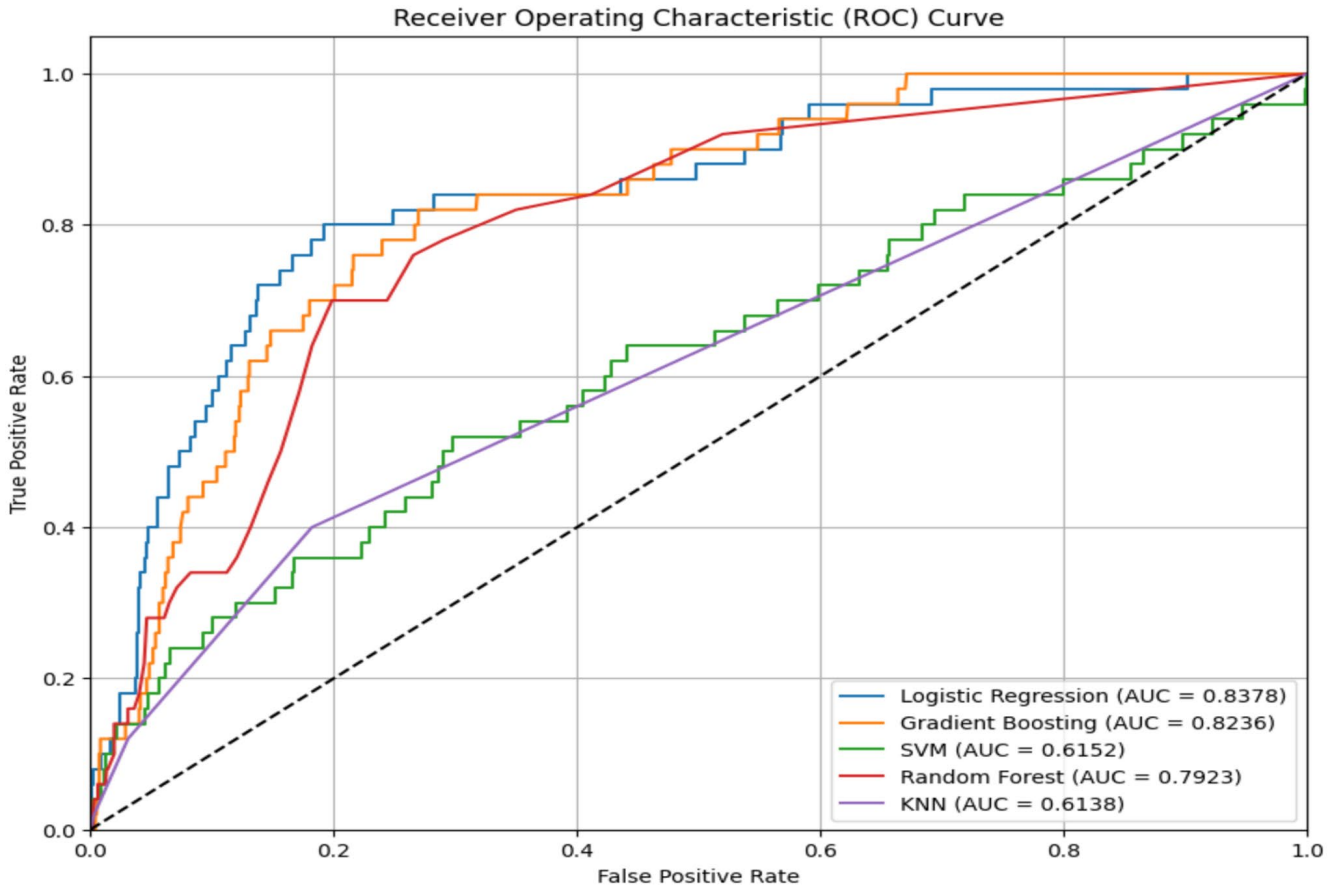
Model ROC curve.

The performance of five supervised learning algorithms was evaluated using five-fold cross-validation. The metrics include mean accuracy, ROC-AUC, and their respective standard deviations. Logistic Regression yielded the highest mean accuracy (0.9511) and a strong ROC-AUC of 0.8362. Gradient Boosting and Random Forest followed closely in both accuracy and ROC-AUC, while SVM and KNN exhibited significantly lower ROC-AUC scores (Figures 2, 3, Table 2). ROC curves (Figure 3) further illustrated that Logistic Regression and Gradient Boosting consistently outperformed other models across various thresholds. The aggregated model performance across evaluation metrics is visualized in Figure 2.

**Table 2:** Model performance metrics.

Model	Accuracy after CV	TN	FP	FN	TP	AUROC score
LR	95.11%	972	0	50	0	0.84
Gradient boosting	94.9%	968	4	49	1	0.82
SVM	95.12%	972	0	50	0	0.60
RF	94.95%	970	2	50	0	0.80
KNN	94.18%	969	3	49	1	0.61

### 4.2 Classification performance (confusion matrix)

**Figure 4:**
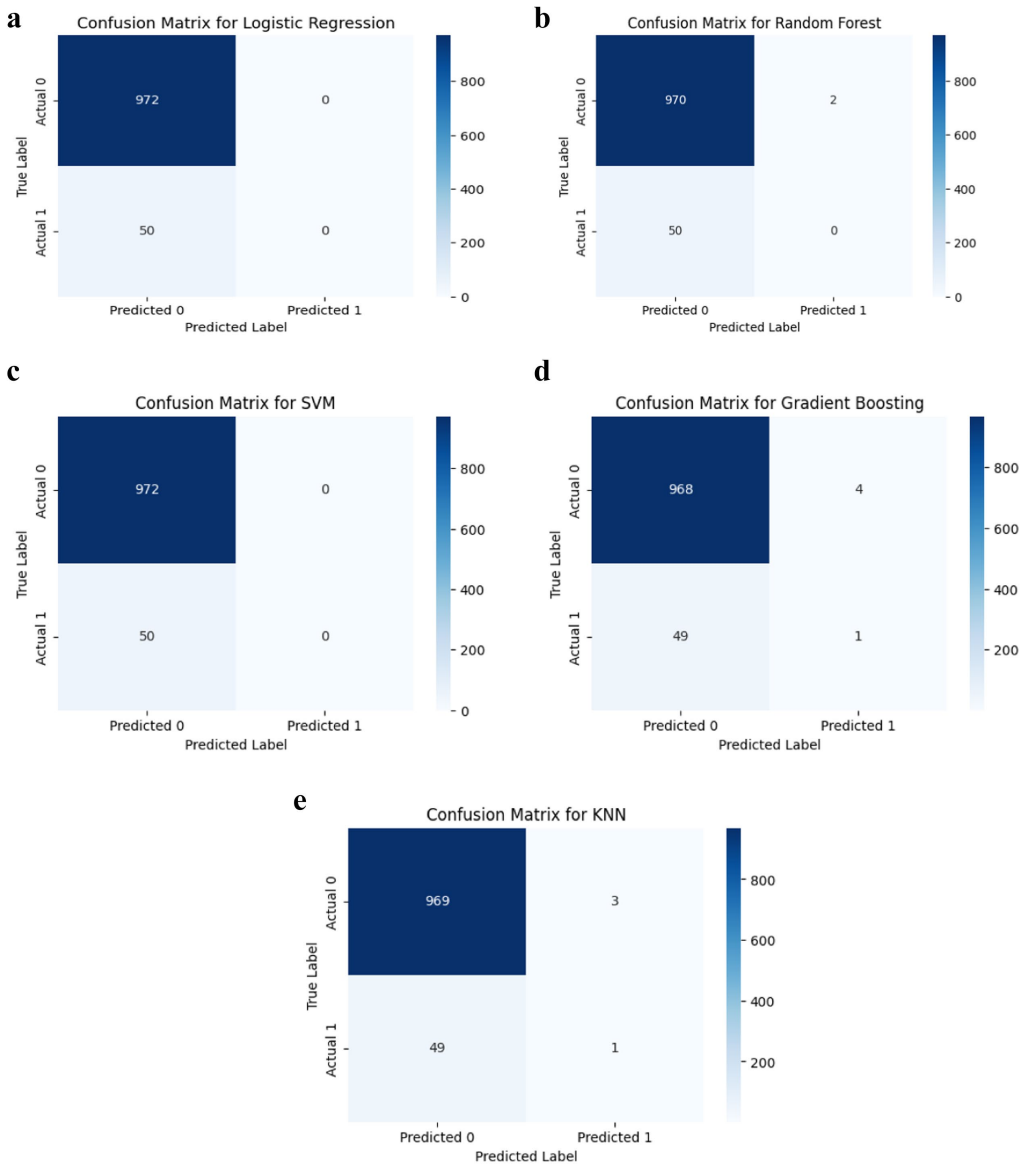
(a) Logistic regression classification performance. (b) Random forest classification performance. (c) SVM classification performance. (d) Gradient boosting classification performance. (e) KNN classification performance.

Despite high accuracy, most models demonstrated limited sensitivity to stroke cases. Logistic Regression, SVM, and Random Forest failed to detect any true positives for stroke (Figure 4), while Gradient Boosting and KNN correctly identified only one stroke case each. This indicates that although the models perform well on non-stroke predictions, they are limited in detecting actual stroke events.

### 4.3 Feature importance

**Figure 5:**
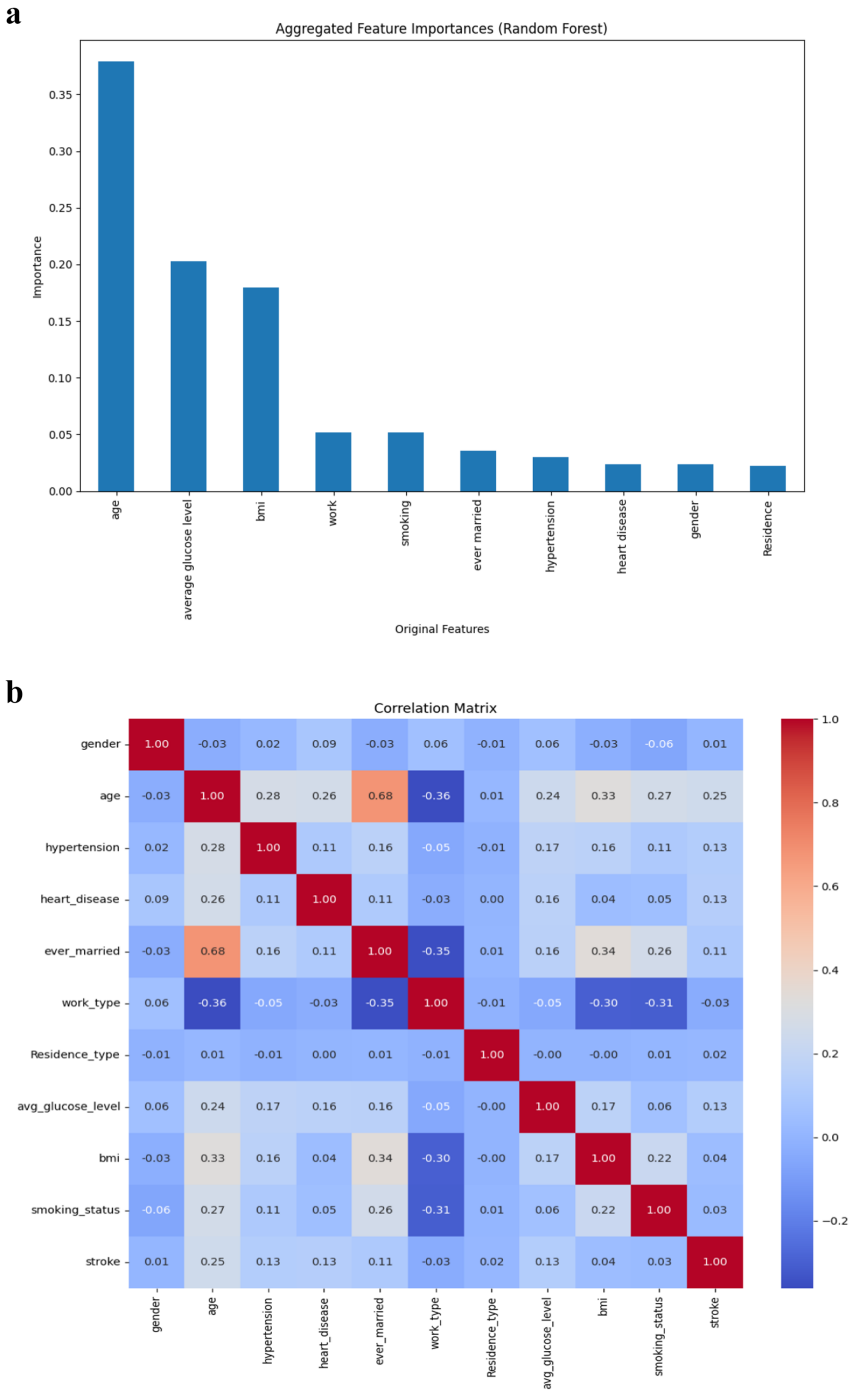
(a) Risk factors (Through Feature Importance). (b) Pearson correlation of features.

Random Forest’s feature importance ranking (Figure 5a) identified age, average glucose level, and BMI as the most influential predictors of stroke. Smoking status and work type showed moderate relevance, while gender, heart disease, and residence type contributed minimally. The correlation matrix (Figure 5b) showed weak to moderate associations between variables and the stroke outcome, affirming the need for multivariate predictive modelling.

### 4.4 Exploratory insights into key stroke predictors

**Figure 6:**
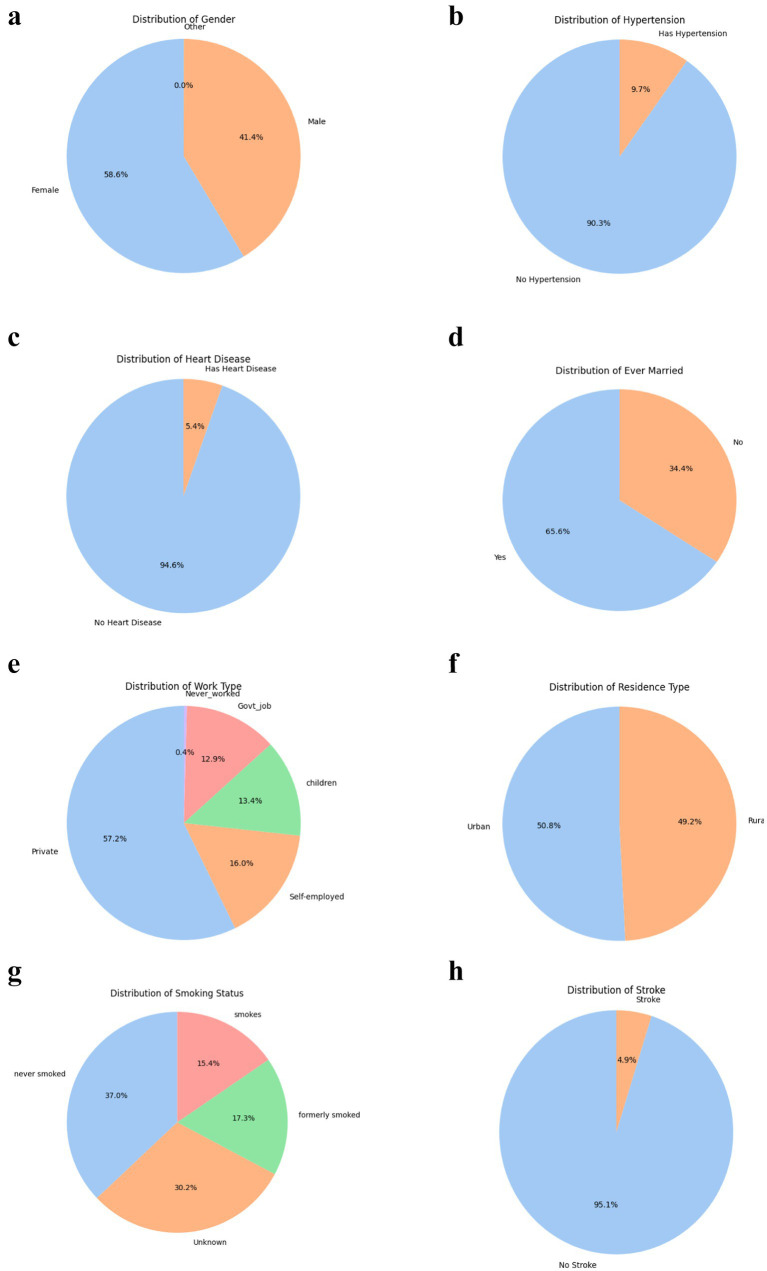
(a) Gender distribution. (b) Hypertension distribution. (c) Heart disease distribution. (d) Ever-married distribution. (e) Work type distribution. (f) Residence type distribution. (g) Smoking status distribution. (h) Stroke distribution.

The dataset reveals insightful patterns in demographic and clinical characteristics relevant to stroke risk prediction, as shown in Figures 6a–h. Female participants constituted 58.6% of the sample, consistent with evidence suggesting women utilize healthcare services more frequently, specifically, studies and report validate that women seek more healthcare and treatment even when excluding maternity-related services (183, 184). Additionally, a 2021 Kaiser Family Foundation study demonstrates that women face significant access and cost-related barriers but remain the higher users of services generally (185–187). These findings reinforce the demographic pattern observed in our data, thereby strengthening the rationale for the observed sample distribution.

However, clinical variables such as hypertension (9.7%) and heart disease (5.4%) were underrepresented in this dataset compared to typical stroke cohorts, where hypertension and cardiac comorbidities are present in over 80% and 50–70% of patients, respectively (188–190). This likely reflects the community-based nature of the dataset rather than a clinically enriched stroke population. This may limit the predictive strength of these factors and highlights a common limitation in using general population datasets for rare event prediction. Similarly, marital status (65.6% married) and work type (57.2% private sector) may act as proxies for social and occupational stressors, recognized contributors to stroke risk (32).

Smoking behavior was diverse, with 15.4% current smokers, 17.3% former smokers, and 37% never smokers, though 30.2% were labelled “Unknown, “introducing missingness challenges. Urban and rural residence were nearly equally split, supporting balanced analysis of geographic disparities in healthcare access (191). Critically, only 4.9% of individuals had experienced a stroke, confirming the rarity of the outcome and the need for class-balancing techniques like oversampling (173). Collectively, these distributions reflect both strengths (e.g., sociodemographic diversity) and limitations (e.g., rare outcome, incomplete data) that must be carefully managed in predictive modelling.

**Figure 7:**
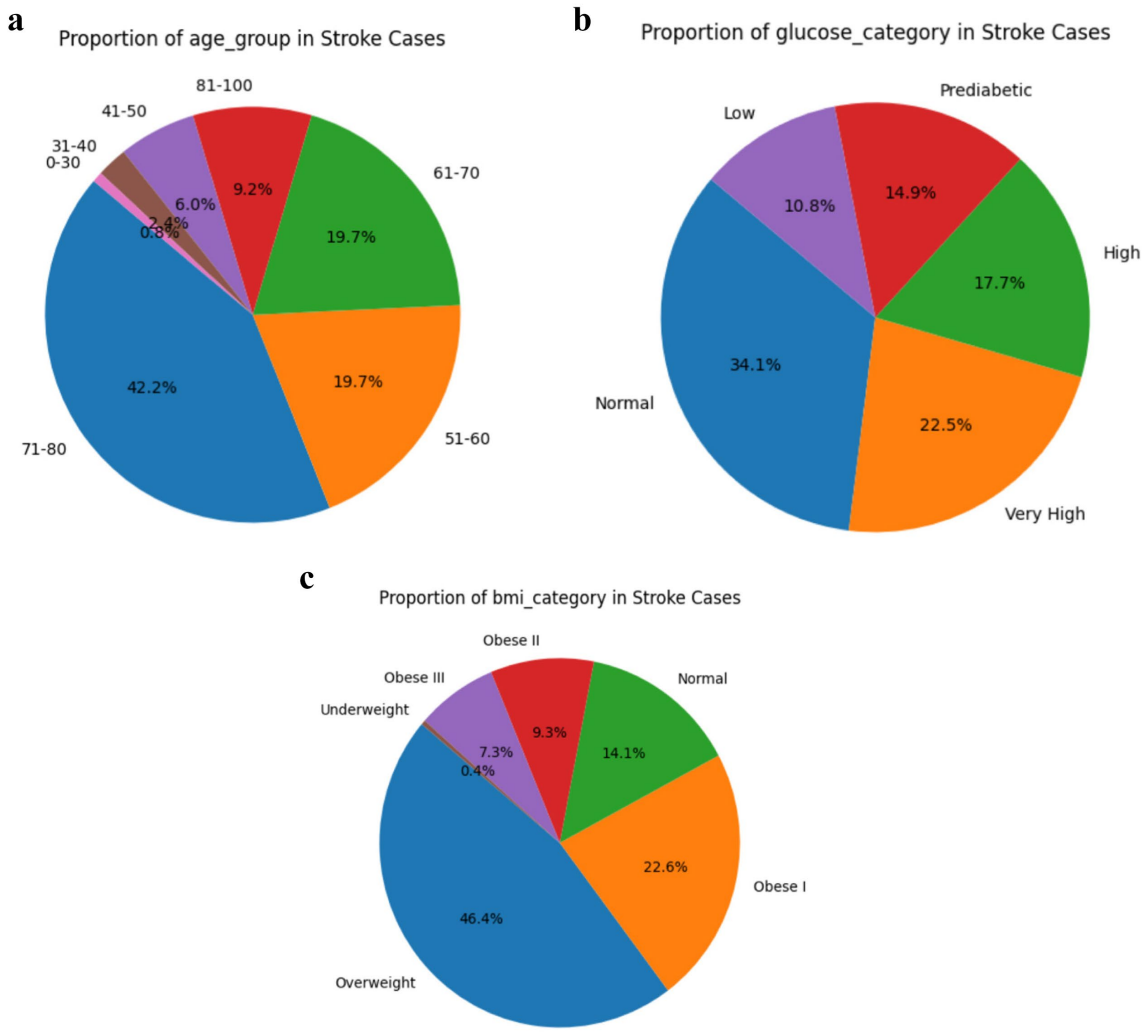
(a) Age group distribution in stroke cases. (b) Glucose category distribution in stroke cases. (c) BMI category distribution in stroke cases.

Lifestyle distributions in Figures 7a–c provide critical insight into the clinical and lifestyle characteristics associated with stroke occurrence, reinforcing the predictive relevance of certain variables identified in this study. Notably, individuals with very high or high glucose levels represent over 40% of stroke cases, highlighting the role of hyperglycaemia and insulin resistance in cerebrovascular risk, a finding that aligns with the Metabolic Syndrome Hypothesis (192, 193). Similarly, more than 70% of stroke cases occur in individuals categorized as Obese I or II, underscoring BMI as a strong modifiable predictor consistent with prior work linking obesity to increased stroke risk due to systemic inflammation and endothelial dysfunction (194, 195). Age also emerged as a dominant factor, with over 80% of stroke cases concentrated in the 51–80 age group, supporting the Framingham Risk Score model which emphasizes age as a primary determinant (196, 197). The distribution of smoking status and work type further reveals that former or current smokers and individuals in private or self-employed work categories represent a substantial portion of stroke cases, suggesting that occupational stress and smoking history are meaningful, albeit secondary, risk indicators, findings echoed in studies on psychosocial risk and vascular health (198–201). These visual trends corroborate the study’s model-based feature importance results, where age, glucose, and BMI ranked highest, thus strengthening the argument for integrating these features into early stroke prediction frameworks leveraging machine learning.

## 5 Discussion

This study aims to develop and evaluate machine learning (ML) models for stroke risk prediction using a combination of demographic, lifestyle, and clinical features. The analysis provides insights into the relative predictive value of different variables, the challenges posed by class imbalance, and the trade-offs associated with various modelling approaches. The findings are contextualized within established clinical frameworks and recent methodological literature, offering both theoretical and practical implications for predictive healthcare.

The demographic structure of the dataset largely mirrors national trends reported in epidemiological studies. Predominantly, 58.6% of participants were female and 65.6% were married, figures consistent with community health surveys.

However, key clinical predictors such as hypertension (9.7%) and heart disease (5.4%) were underrepresented in the dataset compared to broader stroke cohorts (202, 203), and this imbalance was addressed using random oversampling, which preserves original data distributions and clinically relevant patterns without introducing synthetic noise (204, 205). This underrepresentation likely contributed to the reduced predictive weight of these factors in the present models, two conditions well-established in the literature as major stroke risk factors (206, 207). Similarly, while smoking, a known stroke risk factor per (32), was included, it ranked lower in feature importance. While 37% of participants had never smoked, 17.3% were former smokers and 15.4% current smokers. Although smoking status did not emerge as a top-ranking feature, its inclusion aligns with both the Framingham risk score and atherosclerotic pathophysiology, which implicate tobacco exposure in endothelial dysfunction and vascular damage (208–210). Lifestyle-related patterns further shaped the modelling outcomes. The inclusion of work type and residence provides a novel view into the social determinants of stroke risk, with 57.2% employed in the private sector, followed by self-employment (16%) and government service (13%), and distinct health implications across occupational categories. These occupational categories may reflect varying levels of psychosocial stress, physical activity, and access to healthcare, each a known contributor to stroke risk (32).

Addressing class imbalance was a central methodological focus. Stroke cases comprised only 4.9% of the dataset, a rate comparable to that found in Fernández et al. (165) and Nguyen et al. (24), who similarly noted the challenge of modelling rare events in health prediction. Random oversampling was employed to balance stroke and non-stroke observations (Figure 2), as it preserves the original feature space without introducing synthetic noise, making it preferable to SMOTE in small or noisy clinical datasets (204). Although random duplication may increase the risk of overfitting, this was mitigated through stratified k-fold cross-validation to support model generalizability. Unlike SMOTE or SMOTE-ENN, which generate synthetic samples and may distort minority class boundaries when clusters are sparse or outliers are present, random oversampling maintains clinical fidelity, a critical factor in medical risk prediction tasks (31, 211).

While random oversampling improved class balance and training conditions, model recall for stroke cases remained critically low. This finding supports ongoing critiques that metrics like accuracy and AUROC may overstate performance in imbalanced medical datasets (173, 212). Despite Logistic Regression yielding 95.11% accuracy and an ROC-AUC of 0.837, it, along with SVM and Random Forest, failed to identify any true positives. This limitation likely reflects not only class imbalance but also the underrepresentation of key clinical features (e.g., hypertension, heart disease), lack of temporal or severity-related variables, and low feature resolution. Enhancing recall in future studies may require integrating longitudinal or temporal data, using cost-sensitive or ensemble-based learning, and fine-tuning classification thresholds to optimize sensitivity while preserving interpretability.

The balanced dataset allowed for model training, a step essential for enabling fairer training conditions and improving sensitivity to stroke cases, which are otherwise underrepresented in model learning, yet model recall remained limited, a finding aligned with (173), who criticized the reliability of accuracy and AUROC in imbalanced datasets. Indeed, despite Logistic Regression achieving 95.11% accuracy and a ROC-AUC of 0.837, its failure, along with SVM and Random Forest, to identify any true positive cases underlines the clinical risk of relying on global metrics alone. Random oversampling was effective in correcting for class imbalance during training but was insufficient to improve model sensitivity in practice. This suggests that while balancing data is necessary, it is not by itself a sufficient solution for rare-event detection. More recent advancements, including cost-sensitive learning and hybrid ensemble methods, have shown superior performance in improving sensitivity and addressing class imbalance in clinical prediction tasks (64, 213).

Beyond class imbalance, algorithmic bias due to underrepresentation of critical clinical predictors, such as hypertension (9.7%) and heart disease (5.4%), was mitigated through stratified k-fold cross-validation, ensuring balanced representation of key features across all training folds. Furthermore, feature importance analysis using Random Forests enhanced interpretability and transparency, helping to monitor whether underrepresented variables contributed meaningfully to predictions. While these steps improved fairness and generalizability, future research should explore data enrichment, reweighting, or transfer learning to further correct structural imbalances in clinical datasets (180, 214).

The predictive models gave excellent performance ranging from 94.18–95.12% accuracy. Performance metrics from five ML algorithms, Logistic Regression, Gradient Boosting, Random Forest, Support Vector Machine (SVM), and K-Nearest Neighbours (KNN), were evaluated using five-fold cross-validation. Logistic Regression yielded the highest average accuracy (95.11%) and a strong ROC-AUC score (0.8362), closely followed by Gradient Boosting and Random Forest (Figure 2, Table 2). ROC curves (Figure 3) reinforced these findings, with Logistic Regression and Gradient Boosting performing consistently well across threshold variations. The comparative performance across all metrics is summarized in Figures 2, 3, 4a–e and Table A1. The study’s results support prior findings by Moulaei et al. (104), who observed that Random Forest often outperform more complex neural models in structured health data. Gradient Boosting performed competitively in terms of AUROC but identified only one true positive case, underscoring the inadequacy of accuracy in evaluating model effectiveness for rare-event predictions. Studies have noted that even ensemble methods need augmentation with cost-sensitive learning to capture minority outcomes effectively (215, 216).

However, despite these favorable metrics, confusion matrices revealed a profound limitation: most models were unable to correctly classify stroke cases. Logistic Regression, SVM, and Random Forest identified zero true positives, while Gradient Boosting and KNN detected only one stroke case each (Figure 5). These results underscore a key limitation in predictive modelling of rare clinical outcomes, namely, that overall accuracy and ROC-AUC may remain high even when recall is critically low. As noted by Saito and Rehmsmeier (173), recall is especially vital in healthcare contexts where false negatives carry substantial clinical risk. Similar limitations were reported by Fernández et al. (165), who demonstrated that conventional performance metrics often obscure the poor sensitivity of models trained on imbalanced medical data.

The confusion matrices demonstrate the limited ability of the evaluated models to detect stroke cases in an imbalanced dataset. Logistic Regression and SVM both classified all 972 non-stroke cases correctly but failed to identify any of the 50 stroke cases (true positives = 0, false negatives = 50). Similarly, Random Forest predicted 970 true negatives and 2 false positives but also missed all stroke cases (true positives = 0, false negatives = 50). In contrast, Gradient Boosting and K-Nearest Neighbours (KNN) showed marginal improvement, each correctly identifying 1 stroke case (true positives = 1, false negatives = 49), with Gradient Boosting yielding 968 true negatives and 4 false positives, and KNN predicting 969 true negatives and 3 false positives. These results underscore the inadequacy of standard classifiers in handling extreme class imbalance, echoing findings from Saito and Rehmsmeier (173) and Fernández et al. (165), and highlight the need for advanced methods such as cost-sensitive algorithms, synthetic sampling, or ensemble approaches to improve sensitivity in stroke prediction.

The top predictors identified in this study, age, average glucose level, and BMI, are strongly supported by both classical frameworks and recent empirical evidence. These features align with the Framingham Stroke Risk Score and the Metabolic Syndrome Hypothesis, which together highlight the central role of demographic and metabolic variables in cerebrovascular risk. Age remains the most robust non-modifiable predictor, with stroke incidence doubling every decade after age 55 (217–220). Elevated glucose and central adiposity reflect underlying metabolic dysfunction, increasing stroke susceptibility through pathways involving insulin resistance, endothelial injury, and systemic inflammation (221–223). Recent studies confirm that metabolic syndrome nearly doubles ischemic stroke risk, reinforcing the predictive validity of these features (224–226). In contrast, variables like hypertension and heart disease showed lower importance, possibly due to their underrepresentation in the dataset or multicollinearity with stronger features. Furthermore, genome-wide association studies (GWAS) have identified loci such as 9p21, PITX2, ZFHX3, FOXF2, and GUCY1A3 as significant contributors to stroke risk and subtype differentiation, underscoring the interplay of genetic, metabolic, and demographic factors in stroke etiology (227). These insights support the future integration of multi-modal data, clinical, metabolic, and genomic, into stroke prediction models for greater accuracy and personalization.

In terms of interpretability, Logistic Regression again proved advantageous, reflecting the findings of (228–230), who emphasized the clinical value of transparent models in decision support. Yet even interpretable models like LR struggled with recall, reaffirming that rare-event prediction demands strategies beyond resampling, potentially involving advanced techniques like threshold adjustment, anomaly detection, or hybrid architectures (231). Among the models, Logistic Regression stood out for its interpretability and performance, which may reflect the linear relationship between several input variables and stroke risk. Gradient Boosting, while less interpretable, showed competitive ROC-AUC values, indicating strong threshold-invariant performance (232). Random Forest, although robust in accuracy, did not improve stroke case detection, highlighting the tendency of ensemble models to prioritize majority class performance without targeted parameter tuning or cost-sensitive design.

Ultimately, this study confirms the feasibility of supervised machine learning for population-level stroke prediction, identifying age, average glucose, and BMI as the most influential risk factors. While Logistic Regression and Gradient Boosting demonstrated strong accuracy and discrimination, their limited recall underscores persistent challenges in predicting rare stroke events. These findings align with broader literature on class imbalance and sensitivity, highlighting the need for more tailored algorithmic strategies, multi-modal datasets, and clinically relevant evaluation metrics. Incorporating genomic data, especially given the growing body of literature on stroke-associated gene loci, may further refine prediction models and support personalized risk stratification (233). Future research should explore ensemble methods and incorporate multi-modal data, such as genomic loci (e.g., *9p21*, *PITX2*), imaging biomarkers (e.g., white matter lesions), and wearable sensor data, to improve model sensitivity, clinical relevance, and real-world utility in stroke prevention and risk stratification (139, 227).

### 5.1 Implications and limitations

From a practical standpoint, this study underscores the potential of machine learning, particularly interpretable models like Logistic Regression, as a complementary tool for early stroke risk screening. However, the consistently low sensitivity across models highlights a key limitation: standard ML approaches, even when well-calibrated, struggle to detect low-prevalence conditions like stroke without specialized techniques. Additional clinical features, longitudinal data, or advanced methods such as ensemble stacking or deep learning may be necessary to enhance recall. Key limitations include reliance on a single dataset, potential measurement errors, and the absence of time-series or imaging data. Future research should prioritize richer, multi-modal datasets and tailored algorithms to improve predictive robustness and clinical relevance. To improve model sensitivity, future work should integrate multi-modal data, such as genomics (e.g., 9p21, PITX2), imaging markers (e.g., white matter lesions), and real-time inputs from wearables, to capture stroke risk more comprehensively (139, 227). Federated learning may enable this integration across clinical settings while preserving privacy (234, 235).

## 6 Conclusion

This study aimed to develop and evaluate machine learning models to predict stroke risk using demographic and clinical attributes. It contributes to stroke ML research by confirming the feasibility of supervised learning for population-level stroke prediction, but it also identifies critical challenges, particularly around imbalance and sensitivity, that align with broader literature. With stroke remaining one of the leading causes of death and long-term disability globally, early identification of at-risk individuals is critical to effective prevention and timely intervention. By applying five supervised learning models, Logistic Regression, Gradient Boosting, Random Forest, Support Vector Machine (SVM), and K-Nearest Neighbour (KNN), the study assessed model performance in terms of accuracy, ROC-AUC, and classification sensitivity.

Among the models, Logistic Regression achieved the highest ROC-AUC (0.8378), indicating superior ability to discriminate between stroke and non-stroke cases across varying thresholds. However, Gradient Boosting and KNN were the only models that successfully identified any actual stroke cases in the confusion matrix, each detecting one true positive. This highlights a crucial trade-off: while Logistic Regression performs well statistically, Gradient Boosting offers better safety implications by reducing the risk of false negatives, a critical factor in stroke care. Feature importance analysis consistently highlighted age, average glucose level, and BMI as the most influential predictors, aligning with established stroke risk models such as the Framingham Risk Score (29, 217, 225) and the Metabolic Syndrome Hypothesis (35). Lifestyle-related factors, including smoking and occupational class, also contributed meaningfully to model predictions, reflecting real-world social and behavioral determinants of health. The practical value of this study lies in its demonstration of how predictive models, particularly Logistic Regression and Gradient Boosting, can support early stroke detection using accessible, non-invasive data. However, the low recall across all models suggests that further enhancements, such as incorporating diagnostic data, using cost-sensitive learning, or applying hybrid ensemble techniques, are necessary to improve clinical applicability. Future studies should integrate real-time diagnostic features with demographic and behavioral data to build more sensitive models capable of minimizing false negatives. Such improvements would better serve the goal of stroke prediction in safeguarding lives through accurate, timely, and interpretable risk assessment.

## Data Availability

Publicly available datasets were analyzed in this study. This data can be found here: https://www.kaggle.com/datasets/fedesoriano/stroke-prediction-dataset.

## Appendix

**Table A1:** Data attributes.

Serial	Attributes	Description	Type
1	Id	Unique identifier	Identifier
2	Gender	“Male,” “Female” or “Other”	Nominal
3	Age	Age of the patient	Nominal (Quantitative)
4	Hypertension	0 = no hypertension, 1 = has hypertension	Categorical
5	Heart disease	0 = no heart disease, 1 = has heart disease	Categorical
6	Ever married	No or yes	Nominal
7	Work type	Children, government job, never worked, private, or self-employed	nominal
8	Residence type	Rural or urban	nominal
9	Average glucose level	Average glucose level in blood	Numerical
10	BMI	Body mass index	numerical
11	Smoking status	Formerly smoked, never smoked, smoke or unknown	Nominal
12	Stroke (target variable)	1 = had a stroke, 0 = no stroke	Categorical (Binary)

Unknown” in smoking status means that the information is unavailable for this patient.
